# Serosurvey of Avian Influenza Viruses (H5, H7, and H9) and Associated Risk Factors in Backyard Poultry Flocks of Lahore District, Pakistan

**DOI:** 10.3389/fvets.2021.631164

**Published:** 2021-03-24

**Authors:** Mamoona Chaudhry, Hamad Bin Rashid, Michael Thrusfield, Mark C. Eisler, Susan C. Welburn

**Affiliations:** ^1^Infection Medicine, Deanery of Biomedical Sciences, Edinburgh Medical School, College of Medicine & Veterinary Medicine, The University of Edinburgh, Edinburgh, United Kingdom; ^2^Deptarment of Epidemiology and Public Health, University of Veterinary and Animal Sciences, Lahore, Pakistan; ^3^Deptarment of Surgery and Pet Sciences, University of Veterinary and Animal Sciences, Lahore, Pakistan; ^4^Royal (Dick) School of Veterinary Studies, College of Medicine and Veterinary Medicine, University of Edinburgh-Easter Bush Campus, Roslin, United Kingdom; ^5^Bristol Veterinary School, University of Bristol, Bristol, United Kingdom; ^6^Zhejiang University-University of Edinburgh Institute, Zhejiang University School of Medicine, Zhejiang University, Haining, China

**Keywords:** avian influenza, backyard, poultry, H9, sero-prevalence, risk factors, Pakistan, zoonosis

## Abstract

Rural poultry constitutes 56% of the total poultry population in Pakistan; however, epidemiological information about avian influenza viruses (AIVs) in backyard poultry flocks is lacking. A cross-sectional survey of villages of Lahore district was conducted from July 2009 to August 2009 using two-stage cluster sampling and probability proportional to size (PPS) sampling to estimate seroprevalence and its associated risk factors. A random selection of 35 clusters from 308 villages of Lahore were considered, and from each cluster, six chickens aged >2 months were selected. A total of 210 serum samples were collected and examined by the hemagglutination inhibition (HI) test for specific antibodies against AIV subtypes H5, H7, and H9. Overall weighted seroprevalence for AIVs was 65.2% (95% CI: 55.6–74.8%), and for subtype H5, H7 & H9 was 6.9% (95% CI: 10.8–23.0%), 0% (95% CI: 0–1.7%), and 62.0% (95% CI: 52.2–71.8%) respectively. However, none of the samples were positive for H7. The average flock size was 17.3 birds, and the main purpose of keeping poultry was for eggs/meat (70.6%, 95% CI: 59.7–81.4). A majority of them were reared in a semi-caged system (83%, 95% CI: 74.5–91.3). Backyard birds were received from different sources, that is, purchased from the market or received as a gift from friends or any NGO, and were 5.7 times more likely to become avian influenza (AI) seropositive than those that were not exposed to these sources (CI 95%: 2.0–716.0). Backyard birds which were received from different sources, that is, purchased from the market or received from friends or any NGO, were 5.7 times more likely to become AI seropositive compared to those that were not (CI 95%: 2.5–18.7). To reduce the risk of AIV in Pakistan, continuous surveillance of backyard poultry would be needed.

## Introduction

With the introduction of intensive poultry production, new breeds, improved biosecurity, and preventive health measures, poultry production has undergone drastic changes globally. In developing countries, however, the adoption of intensive production has been more restricted due to the cost of infrastructure to maintain biosecurity for birds, the cost of rearing quality hybrid chicks, and the cost of providing balanced feed and quality veterinary care (1). In these countries, most poultry is categorized as “family poultry,” small-scale poultry kept by households using family labor and locally available feed resources when available. Poultry may run freely within the household/compound and scavenge much of their food while getting supplementary food from the householder. Flock size rarely exceeds 100 birds of unimproved or improved breeds. Labor is unsalaried and drawn from the family household (2). In developing countries, poultry keeping makes a major contribution toward the provision of both income and livelihood for many rural households (3). Backyard poultry is rarely the sole means of livelihood for the family; it is a complementary farming activity that contributes to the overall wellbeing of the household. Poultry keeping is a major income-generating activity and provides a valuable source of protein in the diet. Poultry also play an important sociocultural role in many societies. Women have an important role in the development of family poultry production (4), and almost all rural and peri-urban families keep a small flock of 5–20 adult chickens, mostly managed by women and children. Profits are normally low, and products are used for home consumption, given as gifts, or offered for religious purposes (1, 3, 5).

The majority of backyard poultry owners may be ignorant of basic biosecurity measures and the potential risk posed by zoonotic diseases to humans. Sick birds may be handled, sold, slaughtered, and consumed without consideration that the infection in cooked chicken could be potentially harmful to humans (3). Avian influenza virus (AIV) appears to affect all sectors of the poultry population in most Asian countries, but its presence in free-range commercial ducks, village poultry, live bird markets, and fighting cockerels seems to be especially significant in the spread of the virus (6, 7).

In Pakistan, every rural family and every fifth family in urban areas are associated with poultry production activities in some manner (8). The average flock size for backyard poultry is 22 birds. Investment in the poultry sector in Pakistan is estimated at 1 billion US$, but the industry faces various management problems such as infectious diseases (AIV). Rural poultry contributes 56% of the total egg production and 25% of the poultry meat. There is a strong preference for eggs and meat from rural poultry, and their market prices are high compared with the commercially produced eggs and meat (9). Although rural poultry constitutes up to 56% of the total egg production and 25% of the poultry meat and there are reports of various outbreaks of AIV subtypes H5, H7, and H9 in commercial poultry from the different parts of the country, the detailed epidemiology of the diseases in backyard poultry is largely unknown (10, 11). The main objective of the current study was to provide accurate estimates of the seroprevalence of AIVs, specifically subtypes H5, H7, and H9, which have been reported in commercial poultry, to identify high risk areas or villages for future surveillance and progressive control of AIV in Pakistan and to characterize the backyard production system in Pakistan.

## Materials and Methods

### Study Design

A cross-sectional survey of backyard poultry in 35 clusters (30 villages) in different union councils (UCs) of Lahore district was conducted from July 2009 to August 2009. The total number of villages/rural settlements is 308 in 150 UCs of Lahore district (12). The target population included all chickens kept as backyard poultry in the villages/rural settlements of the Lahore district of Pakistan. The study population included healthy chickens of >2 months of age that were reared in backyards of village households of Lahore district for egg or meat production, and the outcome of interest was whether any chicken was diagnosed as either positive or negative for AIV (H5, H7, and H9) by the hemagglutination inhibition (HI) assay. A complete list of enumerating units (the total number of birds in households) in different villages of Lahore was unfortunately not available. Only a list of villages with an estimated total population of backyard birds was available. This information was gathered by the Department of Livestock and Dairy Development, Punjab, for a livestock census survey of Lahore district, in 2000 (12). The census data were used as the basic data frame to draw samples for the current study, and two-stage cluster sampling with probability proportional to size (PPS) with replacement (WR) was selected since no sampling frame of enumerating units was available (13). Villages were included as clusters or a primary sampling unit (PSU) at the first stage, and chickens of >2 months of age were taken as elementary units at the second stage of the two-stage cluster sampling method.

### Sample Size

The sample size was calculated using the C-Survey software, version 2.0 (14). To determine the sample size, the estimated proportion with an attribute was conservatively kept as 50% (13). The precision of the prevalence estimate was set at ±10% at the 95% level of confidence, and the value of design effect (DE) was 2 based on the estimates reported by Bennett et al. (13) and Otte and Gumm (15). Thus, the total number of clusters required for this survey was 35. The elementary units required per cluster were fixed, i.e., six birds per cluster were selected systematically without replacement. The total sample size for the proposed study, therefore, was 210 backyard birds from 35 clusters (30 villages). Four villages (Chung, Dhullam Kurd, Dhullam Jullian, and Kungh Sharif) of Lahore district were sampled more than once, and each selection from each village was considered as the PSU, as the sampling method was WR (Supplementary Table 1). All birds in a village were treated as a single flock since they were kept under similar conditions. Blood samples were collected from the captured birds that were systematically selected. The central point of the village (e.g., mosque) was selected as a starting point, and survey sampling was started on a random side by using the “Spin the Pen” method (16). While moving through the village, every fifth healthy bird observed was selected, and permission was requested from the owner to collect blood from the brachial vein (17) until the desired number of samples was obtained.

### Laboratory Analysis

Blood, collected from the brachial vein of the selected birds, was immediately transferred to serum separator tubes, and serum samples were allowed to clot by slightly inclining the tubes at room temperature (22–25°C) before refrigeration. The separated sera were transferred to labeled screw-capped cryotubes and stored at −20°C. Serum samples were thawed once and examined within 1 month post-collection by the HI test for specific antibodies to AIV subtypes H9, H5, and H7 according to the OIE Diagnostic Manual (18). The circulation of these subtypes of AIV has been reported from Pakistan previously; therefore, the samples were only screened for these subtypes (10, 11). The HI test was performed using four hemagglutinin units of virus antigen (4HAU) and 1% chicken erythrocytes diluted in phosphate buffer saline (PBS). Serial 2-fold serum dilutions in PBS were mixed with 25 μl of the virus (4HAU), and then, 25 μl of washed chicken red blood cells were added and mixed well. After that, the plates were incubated for 30 min at room temperature (20–25°C). The HI titer was calculated as the reciprocal of the highest dilution of serum that inhibited the hemagglutination of the chicken erythrocytes. Samples with titers ≥1:16 were considered as positive. Reference antigens [A/turkey/wisconsin/68 (H5N9); A/Chicken/Italy/1067/99 (H7N1); H9N2 (Middle East origin), Merial Italia S.p.A., Noventa, Italy] and antibodies (VLDIA042 HAR-INFH5, VLDIA043 HAR-INFH7, VLDIA150 HAR-INFH9, GD, Animal health service, Deventer, The Netherlands) for H5, H7, and H9 were used to test the serum samples. All hypodermics and biological waste were disposed of properly. The work was done at the Grand Parent Poultry Laboratory, Lahore, Pakistan and the University of Veterinary and Animal Sciences, Lahore, Pakistan.

### Data Collection

A questionnaire was designed based on an extensive review of the previous literature and biological plausibility of the risks (19–27) and was pretested in five villages with 10 respondents in the same area in which the sampling was to be undertaken. The questionnaire included 34 closed and semi-closed questions. Information on flock size and type, the purpose of keeping backyard birds, qualifications and profession of the owner, other animals maintained with the backyard birds, and management practice (questionnaire in Supplementary Material) was collected from the owner of each selected bird in a face-to-face interview after receiving their consent. All owners permitted to collect blood samples from their birds and answered all questions in the questionnaire. Permission to conduct the survey was obtained from the Livestock and Dairy Development Department of Punjab, Pakistan.

### Statistical Analysis

Locations of the villages and households owning the selected birds were recorded with a hand-held global positioning system (GPS; Garmin, Olathe, KS, USA). The data collected during the survey from questionnaires were stored in EpiData Entry version 3.1 (28) and were validated by rechecking the computerized data and by matching it to the hard copy. The stored data were exported in dBase and the Excel format for further processing and analysis in ArcGIS 10 (Geographical Information System, ESRI System, Redlands, CA, USA), and a statistical analysis was undertaken in the R-statistical computing environment version 2.14.0 (29).

The weighted proportion estimates with 95% CIs of the overall seroprevalence were computed by the svy function using the survey package in R (30). Point estimates of the weighted means, percentages for each characteristic of interest, and 95% CIs were also calculated (30). Within each village, prevalence point estimates were computed by the epi.conf function in the epiR package using an exact method for CI calculation.

About 34 potential risk factors were analyzed for association with an outcome by measuring the odds ratio (OR) (31). The survey-weighted logistic regression model was used to fit into the univariable and multivariable models, and seroprevalence ORs with 95% CI for each explanatory variable were calculated using the survey package in the R software (30). All independent variables associated with the seroprevalence of AIV were initially screened with the chi-square test in univariable analysis, and all variables with *p* < 0.25 were included in the multivariable logistic regression model by using a forward stepwise variable selection strategy (31, 32). For building the final model, variables with *p* ≤ 0.05 based on the Wald statistic (or the log-likelihood ratio test for categorical variables with 3 or more levels) were retained in the model. Collinearity among the selected variables was tested using the Spearman's rank correlation (33). If there was a strong positive correlation (ρ > 0.5) between the variables, the more clinically important and biologically plausible variable from pairs of correlated variables was chosen for the multivariable model.

### Spatial Data and Analysis

A paper map of the rural settlements of Pakistan (scale 1:5,000,000) was digitally scanned from the Atlas of Survey of Pakistan published in 2002 by the Survey of Pakistan Office, Rawalpindi (34), and a map showing town boundaries and UC boundaries of Lahore district was downloaded from the website of the City District (http://www.lahore.gov.pk/city-government/lahore-map.aspx). These maps were georeferenced using ArcGIS 10. Point (dot or location) maps and graduated pie maps of the spatial distribution of villages and premises of backyard poultry birds in different UCs of Lahore district were generated (35, 36).

## Results

**Figure 1:**
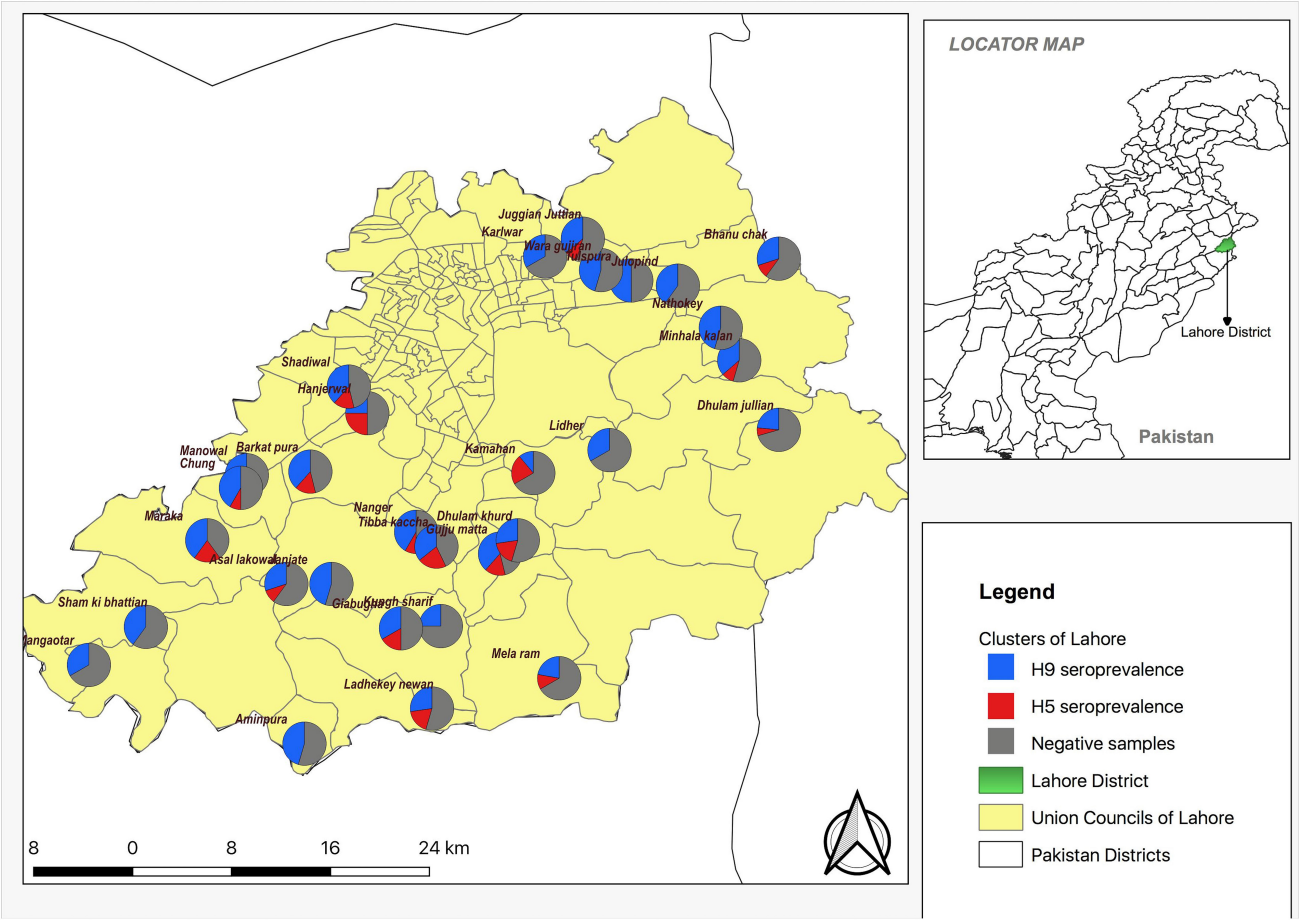
Spatial distribution and village (cluster) level seroprevalence of H9 and H5 in backyard poultry in Lahore district.

A total of 144 sera were positive for AIVs (H5, H9, or both) using the HI test from 210 samples and, among these, 134 serum samples were positive for subtype H9, 38 were positive for subtype H5 A, and 27 samples had antibodies to both subtypes (Figure 1). The distribution of HI titer against subtypes H9 and H5 are presented in Table 1. There were no positive samples for the H7 subtype.

**Table 1:** Distribution of antibody titers against avian influenza virus (AIV) subtypes H5 and H9 in the hemagglutination inhibition (HI) test.

**HI dilution**	**No (%) [95% CI]**	
	**Subtype H5**	**Subtype H9**
**≤**1:8	172 (81.90)	76 (36.19)
1:16	26 (12.38)	10 (4.76)
1:32	8 (3.80)	23 (10.95)
1:64	3 (1.42)	28 (13.33)
1:128	1 (0.47)	23 (10.95)
1:256	0 (0)	15 (7.14)
1:512	0 (0)	21 (10)
1:1,024	0 (0)	9 (4.28)
1:2,048	0 (0)	5 (2.38)
≥1:16	38 [16.9% (10.8–23.0)]	134 [62.0% (52.2–71.8)]

The spatial distribution of positive samples indicated that all 30 villages were seropositive for H9 antibodies while 21 villages were seropositive for H5. Coinfection with H9 and H5 occurred in 18 villages (Figure 1). The overall seroprevalence of AIVs, subtypes H5, H7, and H9, was 65.2% (95% CI: 55.6–74.8%); 16.9% (95% CI: 10.8–23.0%), 0% (95% CI: 0–1.7%), and 62.0% (95% CI: 52.2–71.8%), respectively, calculated by a two-stage cluster analysis with PPS (Table 2). The highest seroprevalence of H5 was in Manowal, Maraka, Hanjarwal, and Tibba Kaccha (50.0%, 95% CI: 11.8–83.2) and the lowest was 0% (95% CI: 0–26.5) in Kungh Sharif, and 0% (95% CI: 0–45.9) in Aminpura, Wara Gujjran, Karlwar, Sham ki Bhattian, Mangaotar, Janjate, Lidher, and Nathokey. The highest seroprevalence for H9 was observed in the Maraka village (100%, 95% CI: 54.1–100), while the lowest seroprevalence for H9 was in Kamahan (16.7%, 95% CI: 0.42–64.1).

**Table 2:** Overall seroprevalence of H5 and H9 estimated from 210 backyard birds in villages of Lahore district (in %).

**AIV subtype**	**Seroprevalence (*****n*** **= 210)**		**Design effect (DE)**
	**Point estimate**	**95% CI**	
AIVs	65.20	55.6–74.8	2.22
H5	16.9	10.8–23.0	1.27
H9	62.0	52.2–71.8	2.08

The average flock size was 17.3 birds (range 1–922 birds), and the main purpose of keeping poultry was for eggs/meat (70.6%, 95% CI: 59.7–81.4). The average number of chickens reared as layers was 3.4 (range 0–50). The most common breed of poultry in the backyard was indigenous Desi (94.0%, 95% CI: 89.2–99.0), followed by a mixture of different breeds (6%, 95% CI: 1.0–10.8). The majority of the birds (83%) were reared in a semi-caged system (95% CI: 74.5–91.3), while 17% of them were kept completely outdoors (95% CI: 8.7–25.5). Most farmers fed their flock with leftover food from home (74.8%, 95% CI: 58.4–91.2), while 25.2% of backyard poultry were fed on leftover food and also scavenged outside the house (95% CI: 8.8–41.6).

The most common source of water intake for birds was both tap water at home and street channel drainage water (60.6%, 95% CI: 41.4–79.8). Some farmers (20.0%, 95% CI: 8.2–31.9) provided only tap water to poultry, while 19.4% of backyard birds used street channels as the main source of water intake (95% CI: 3.3–35.4).

Out of 34 potential risk factors, six variables (the profession of farmers, buying adult birds, the source of birds, selling birds or eggs, disposal of dead birds by the farmer, disinfection of backyard) were excluded from analysis due to insufficient (zero cell value) values in a 2 × 2 table. A total of nine variables showed an association (*p* < 0.25) with the seroprevalence of AIVs in backyard poultry in univariable logistic analysis using the chi-square test. However, two variables (the presence of wild birds in the vicinity and pet animals visiting neighboring commercial farms) were excluded due to collinearity (ρ > 0.5), and seven variables, which were biologically more plausible, were retained in the analysis (Table 3). The final weighted logistic regression model identified two variables out of the initial seven as potential risk factors for AIV in these backyard birds (Table 4). The source of birds (hatched at home vs. other sources) was identified as a risk factor (OR: 5.7; CI 95%: 2.0–16.0, *p* = 0.019). Backyard birds kept in close vicinity of live poultry retail shops were 6.9 times more likely to be AIV seropositive compared to those that were not (CI 95%: 2.5–18.7, *p* = 0.003).

**Table 3:** Results of univariable analysis with potential risk factors associated with the seroprevalence of AIVs in backyard poultry of villages of Lahore district.

**Potential risk factors**	**Response level**	**AIVs result**		**Odds ratio (OR)**	**95% confidence interval for OR**	***P*-value**
		**Negative**	**Positive**			
Source of birds	Hatched at home	18	16	1.16	0.86–1.56	0.02
	Other/gift etc.	48	128			
Access to veterinary hospital	No	58	139	0.36	0.17–0.77	0.013
	Yes	8	5			
Decreased production in birds (eggs & weight)	No	39	61	2.31	1.17–4.57	0.022
	Yes	27	83			
Sharing feed with wild birds	No	9	6	3.96	1.25–12.62	0.027
	Yes	57	138			
Poultry farm workers visiting village	No	34	96	0.49	0.24–0.99	0.057
	Yes	32	48			
Farm vehicle visiting farm	No	28	88	0.44	0.22–0.86	0.024
	Yes	38	56			
Proximity of backyard to live bird retail shops	No	60	112	3.53	1.10–11.32	0.043
	Yes	6	32			

**Table 4:** Results of final model with potential risk factors associated with the seroprevalence of AIVs in backyard poultry of villages of Lahore district.

**Potential risk** **factors**	**Response level**	**Odds ratio (OR)**	**95% confidence interval for OR**
Source of birds	Hatched at home	5.7	2.0–16.0
	Others/gift etc.		
Proximity of backyard to live bird retail shops	No	6.9	2.5–18.7
	Yes		

## Discussion

Backyard poultry infected with AIV can pose a risk for the introduction of AI into commercial poultry (19). In the current study, we estimated the seroprevalence of AIVs in backyard poultry flocks of Lahore district. The overall weighted seroprevalence of AIVs was 65.2% (95% CI: 55.6–74.8%). The present study estimate is higher than an estimate from Bangladesh (23% of flocks and 20% of chickens). This difference in the estimate may be due to a sampling strategy or low circulation of AIVs in Bangladesh (20).

All villages sampled were seropositive for H9, and the overall weighted seroprevalence of H9 was 62% (95% CI: 52.2–71.8%). Various studies from neighboring countries have reported slightly higher estimates of seroprevalence (73 and 81.6% in Iran) in backyard poultry, which suggests endemicity of H9N2 viruses in the region (37, 38). In the current study, the seroprevalence of H5 was 16.9% (95% CI: 10.8–23.0%), which is close to the seroprevalence estimates reported from Bangladesh (9.82%) and Vietnam (17.5%) (26, 39). Backyard birds had not been vaccinated against AI, and the antibody titers represented previous exposure to naturally circulating virus and confirmed that low pathogenic AIVs, H9 and H5, are circulating in backyard poultry in villages of Lahore, Pakistan. The variation in the estimates of seroprevalence of H9, H5, and H7 in our survey could be attributed to the pathogenicity of these subtypes. H5 and H7 have high mortality (40) and chicken infected with these subtypes usually die; hence, the number of seropositive birds was low. On the contrary, H9 is a low pathogenic subtype with endemic status in Pakistan (41), enabling repeated reinfections of the same birds and increasing seroprevalence (42). Furthermore, due to extensive biosecurity measures and the presence of inactivated vaccines in commercial poultry (11, 43), subtypes H5 and H7 have not been reported in commercial poultry in Pakistan since their last outbreak in 2008 (44), which might be attributed to their low or negligible seroprevalence in our survey.

Our results showed that the coinfection of H5 and H9 was present in 18 villages. A total of 27 samples from the backyard birds had antibodies to both the subtypes. The co-circulation of H9N2 and HPAI H5N1 viruses, along with their ability to mutate through antigenic shift and drift, may produce novel viruses with pandemic potential (45). Inter-subtype reassortment between the co-circulating H9N2 virus and the highly pathogenic H5N1 virus has been detected in China (46), Pakistan (47), Bangladesh (48), and Cambodia (49).

Most surveys were conducted to estimate the seroprevalence of AI in Pakistan and have not been incorporated due to lack of adequate study design to avoid bias. In the present study, PPS sampling was used to ensure that unbiased prevalence estimates were obtained. Recently, Henning et al. (26) also reported that bird-level prevalence using a multi-stage sampling design accounts for clustering and sampling fractions.

Characteristics of the backyard poultry production system vary with the socioeconomic and cultural heritages of the developing countries, but most are essentially similar. The major purpose of rearing birds was to get eggs and meat for personal use (70.6%). It has been observed globally that in more than 90% of the cases, backyard birds are kept for egg production for household consumption (25, 27). However, in Bangladesh, farmers reported the purpose of keeping birds as a cash source and as a protein source (50). Contrary to this data, the data from other countries with high income showed that the farmer ranked hobby/fun as the highest reason for keeping backyard birds (22).

In Pakistan, the most common breed is indigenous Desi. Farooq et al. (51) reported that the indigenous Desi birds dominated the flocks (10.2 birds) followed by Fayoumi (6.76) in Pakistan. This study also showed that the common breed in the backyard was indigenous Desi (94%). Farmers prefer to keep the indigenous Desi breed due to its greater capacity to survive and adapt to scavenging management systems (51). The average size of flock reported in the current study (17.3 birds/flock) is very close to estimates of the livestock census. Various studies from Pakistan have reported a varying size of flock ranging from 22 to 26 birds (51, 52), while other studies from Thailand, Bangladesh, and Chile reported a flock size ranging from 10 to 37 birds (25, 50, 53).

In this study, the majority of farmers reared their backyard birds in a semi-caged system (83%, 95% CI: 74.5–91.3). Other studies also observed that the majority of farmers prefer to rear birds in semi-caged facilities (25, 27, 51). Outdoor backyard flocks may be more at risk for the introduction of AI strains of either high or low pathogenicity (54). Nooruddin et al. (39) also reported that chickens reared under a semi-scavenging system in Bangladesh were allowed to scavenge with ducks in the yard and in the crop fields near to water reservoirs where domestic ducks, wild ducks, and migratory birds were also present. This may contribute to the natural infection of the native chickens. Terregino et al. (55) indicated the backyard poultry farming system as being high risk for AI introduction, primarily due to many free-range holdings. The availability of food attracts wild birds and results in intermingling and in the deposition of droppings.

The results of a logistic regression analysis confirmed that the main risk factors for the infection of backyard flocks with AI were the “source of bird,” i.e., hatched at home vs. other sources and birds that were kept in a backyard vs. close to a live poultry retail shop. Usually, farmers keep birds that are hatched at home; very few purchase birds from local markets or receive them as a gift from friends, and sometimes private NGOs provide free birds to women in villages. Birds purchased from local live bird markets have been previously identified as a risk to backyard poultry for infection with AIVs (56).

Our study showed that backyard poultry birds are usually raised in a unit with a semi-caged system, i.e., they roam around the village area and have access to live bird retail stalls in their vicinity, which may increase the risk of infection in backyard poultry. Contact of backyard birds with live bird markets and wild birds has been identified as a potential risk factor of AIV in backyard poultry (19, 27, 57). Live bird retail stalls pose a continuous threat due to the mixing of birds from various sources and poor sanitary conditions in stalls (58). Awareness of the owners to reduce the free-range rearing of poultry can limit the spread of AIV among backyard poultry. Current findings can be generalized to similar poultry rearing systems in other low- and middle-income countries.

The main limitation is that our study was a cross-sectional survey which only estimates a parameter at a certain point in time. The second limitation was that a more precise sample design (simple random sample) could not be defined due to the lack of a sampling frame. Another limitation of this survey was that only the serological status of the birds was tested; this can only detect antibodies against any previous exposure of the bird to the virus and does not detect live virus. A more detailed longitudinal study for the detection of live virus would better monitor the subtype of live virus circulating in the backyard birds. Neuraminidase subtyping of samples was not done due to financial constraints.

In conclusion, the result of the current study confirmed the presence of antibodies to H9 and H5 in villages, which demonstrate the exposure of chickens to circulating AIV viruses. Based on these results, regular surveillance in villages or peri-urban areas is recommended. To reduce the risk of the spread of AIV in Pakistan, continuous surveillance of backyard poultry would be needed because these birds are at a higher risk of contracting infection due to the free-range system. The presence of AIV in these birds also poses a threat to human health because these birds are in frequent contact with farmers/handlers and interspecies transmission can occur (59). Backyard birds are a vital protein resource for the rural population of Pakistan; it might not be possible to prevent the rearing of these birds. The knowledge and status of AIV in these birds can be used to help devise control strategies for the containment of the spread of AIV in the poultry production system.

## Data Availability Statement

The raw data supporting the conclusions of this article will be made available by the authors, without undue reservation.

## Ethics Statement

Ethical review and approval was not required for the animal study because informed consent for all participating owners of backyard poultry was taken prior to the sampling and data collection. Briefly the objectives of the study and voluntarily participation were explained and they were invited to participate. Only farmers who consented voluntarily were included and interviewed. A trained veterinarian or para veterinarian collected the blood samples from birds. The manuscript does not contain any clinical studies or patient data.

## Author Contributions

SW, ME, MT, and MC contributed conception and design of the study, data analysis, interpretation, read, revised, and approved the submitted version of the manuscript. MC and HR collected the data, organized and carried out statistical analysis, and prepared the manuscript. SW, ME, and MT supervized the proposal preparation, presentation, and data collection. ME and SW contributed equally. All authors contributed to the article and approved the submitted version.

## Conflict of Interest

The authors declare that the research was conducted in the absence of any commercial or financial relationships that could be construed as a potential conflict of interest.
