# Supplementary material for: Serosurvey of Avian Influenza Viruses (H5, H7, and H9) and Associated Risk Factors in Backyard Poultry Flocks of Lahore District, Pakistan
Source: Front Vet Sci. 2021 Mar 24;8:631164. doi: 10.3389/fvets.2021.631164 (PMC8024624; doi:10.3389/fvets.2021.631164)
Supplement: Supplementary file 1 [file Data_Sheet_1.docx]

Supplementary Material

# Supplementary Table

Table. Details of villages selected as clusters with total population of backyard poultry and number of birds selected as enumeration units

| Serial No | Villages/cluster | Union Council | Population size | No. of selected cluster | No. of listing unit |
| --- | --- | --- | --- | --- | --- |
| 1 | Lidher | Lidher | 491 | 1 | 6 |
| 2 | Jullopind | Jullopind | 624 | 1 | 6 |
| 3 | Bhanu Chak | Wagha | 182 | 1 | 6 |
| 4 | Barkatpura | Ali Razabad | 134 | 1 | 6 |
| 5 | Chung | Chung | 2160 | 3 | 18 |
| 6 | Manowal | Chung | 524 | 1 | 6 |
| 7 | Hanjarwal | Niazbaig | 927 | 1 | 6 |
| 8 | Shadiwal | Niazbaig | 1832 | 1 | 6 |
| 9 | Minhalakalan | Minhala | 1037 | 1 | 6 |
| 10 | Nathokey | Minhala | 506 | 1 | 6 |
| 11 | Dhullam Jhullian | Padhana | 220 | 2 | 12 |
| 12 | Dhullam khurd | Dhullam khurd | 749 | 2 | 12 |
| 13 | Melaram (keet) | Jhulkay | 287 | 1 | 6 |
| 14 | Nanger | Kaccha | 340 | 1 | 6 |
| 15 | Tibbah Kaccha | Kaccha | 235 | 1 | 6 |
| 16 | Gajjumata | Kahna | 421 | 1 | 6 |
| 17 | Kamahan | Kamahan | 1121 | 1 | 6 |
| 18 | Mangaotar | Manga | 874 | 1 | 6 |
| 19 | Maraka | Maraka | 998 | 1 | 6 |
| 20 | Shamkay Bhattian | Sultan kay | 491 | 1 | 6 |
| 21 | Jhuggian jatian | Lakhodher | 89 | 1 | 6 |
| 22 | Karulwar | Lakhodher | 487 | 1 | 6 |
| 23 | Wara gujjran | Lakhodher | 388 | 1 | 6 |
| 24 | Tulspura | Manawan | 210 | 1 | 6 |
| 25 | Giabagha | Giabagha | 417 | 1 | 6 |
| 26 | Janjate | Giabagha | 432 | 1 | 6 |
| 27 | Kungh sharif | Giabagha | 255 | 2 | 12 |
| 28 | Ladhkey newan | Ladhkey newan | 401 | 1 | 6 |
| 29 | Asal Lakhowal | Rai | 180 | 1 | 6 |
| 30 | Aminpura | Raiwind | 644 | 1 | 6 |

# Questionnaire for collection of information about characteristics of backyard poultry system

Questionnaire code: _____________________ Questionnaire No._________

Date: _______________________________ Village code _____________

Farmer’s name: ____________________

Geographical Location:

| Region | Latitude | Longitude |
| --- | --- | --- |
|  |  |  |

## Farmer’s Profile:

1. Premises type: (please tick appropriate box......................................Single family 🞏 Joint family 🞏

2. Qualification: (Please circle one only)................................................................................................

……………………..Illiterate 🞏 primary 🞏 middle 🞏 secondary 🞏 higher secondary 🞏 graduate 🞏

3. Family size: _______________________ (No.)

4. Major source of income..............................................................................................................

…………...........Labor 🞏 agriculture and livestock 🞏 Employment 🞏 business 🞏 poultry farming 🞏

## Poultry Profile

5. Purpose of keeping backyard poultry (Please circle)................ For egg or meat 🞏 hobby 🞏 both 🞏

6. Which type of flock do you keep? (Please circle one only):.........................................Desi 🞏 mix 🞏

7. Total no. of birds kept ____________________________ (No.)

## Management Practices and Biosecurity

8. Type of rearing system (Please circle one only):.....................Semi cages 🞏 completely outdoor 🞏

9. Type of feed used (Please circle one only)......................................Leftover 🞏 scavenger 🞏 mix 🞏

10. Type of watering system (Please circle one only):............. Tap water 🞏 street channels 🞏 both 🞏

11. How you get the birds for flock?....................................Hatched at home 🞏 hawkers/gifts/other 🞏

12. Do you buy adult birds (reproductive age)? (Please circle one only).......................... Yes 🞏 No 🞏

13. If yes from where do you get them? (Please circle only one)........Commercial supplier 🞏 other 🞏

14. Have you vaccinated these birds? (Please circle one only)......................................... Yes 🞏 No 🞏

15. Do you sell your birds/eggs? (Please circle one only).................................................. Yes 🞏 No 🞏

16. Is there any commercial farm/hatchery/feed mill near your village?........................... Yes 🞏 No 🞏

17. How far is that from your premises (Km)? _________________________

18. Do you have access to veterinary services? (Please circle one only)........................... Yes 🞏 No 🞏

19. How far is that practice (Km)? ____________________________

20. Does anyone in your family work for a commercial poultry production system or poultry processing system? (Please circle one only)....................................................................... Yes 🞏 No 🞏

21. Do you keep backyard flock and pet birds in the same vicinity? (Please circle one only).................

…………………………………………………………………………………………….. Yes🞏 No🞏

22. Did your birds have sudden decreased production not related to molting (reduced egg laying or hatching rate, no weight gain)? (Please circle one only).................................................... Yes 🞏 No 🞏

23. How you dispose of your dead birds? (Please circle all that are appropriate)........................

……………………………………………….. Buried 🞏 burn 🞏 sold 🞏 left open for scavengers 🞏

24. How often you clean your backyard premises?....................................Regularly 🞏 not regularly 🞏

25. Do you use any disinfectant for cleaning? (Please circle one only)..............................Yes 🞏 No 🞏

26. Is there any pond/canal/stream near your house? (Please circle one only)...................Yes 🞏 No 🞏

27. Have you seen any wild bird around your vicinity? (Please circle one only)...............Yes 🞏 No 🞏

28. Did they share feed and water with your birds? (Please circle one only).....................Yes 🞏 No 🞏

29. Do you visit the neighboring commercial farms? (Please circle one only)..................Yes 🞏 No 🞏

30. Do your birds visit neighboring commercial farms? (Please circle one only).............Yes 🞏 No 🞏

31. Do your pet animals visit neighboring commercial farms? (Please circle one only)....Yes 🞏 No 🞏

32. Have the poultry farm workers visited the village? (Please circle one only)................Yes 🞏 No 🞏

33. Did the farm vehicle visit the village? (Please circle one only)................................... Yes 🞏 No 🞏

34. Do you have any live bird market stall near your house?............................................ Yes 🞏 No 🞏
